# Breast Cancer Screening Interventions for Arabic Women: A Literature Review

**DOI:** 10.1007/s10903-013-9902-9

**Published:** 2013-08-23

**Authors:** Tam Truong Donnelly, Jasmine Hwang

**Affiliations:** 1 University of Calgary-Qatar, Doha, Qatar; 2 Community Health Sciences, Faculty of Medicine, University of Calgary, Calgary, AB Canada; 3 Faculty of Nursing, University of Calgary, 23 Edgeland Close NW, Calgary, AB Canada

**Keywords:** Arab, Breast neoplasms, Breast cancer early detection, Cancer intervention and evaluation

## Abstract

Similar to other Middle Eastern countries, breast cancer is the most common cancer among women in Qatar with increasing incidence and mortality rates. High mortality rates of breast cancer in the Middle Eastern countries are primarily due to delayed diagnosis of the disease. Thus screening and early detection of breast cancer are important in reducing cancer morbidity and mortality. With the aim of updating knowledge on existing interventions and developing effective intervention programs to promote breast cancer screening in Arabic populations in Qatar, this review addresses the question: What interventions are effective in increasing breast cancer knowledge and breast cancer screening rates in Arabic populations in Arabic countries and North America? Systematic literature review was performed to answer the proposed question. As the result of the search, six research studies were identified and appraised. From the findings, we infer several insights: (a) a language-appropriate and culturally sensitive educational program is the most important component of a successful intervention regardless of the study setting, (b) multi-level interventions that target both women, men, health care professionals, and/or larger health care system are more likely to be successful than single educational interventions or public awareness campaigns, and (c) more vigorous, personal and cognitive interventions that address psychosocial factors are likely to be more effective than less personal and informative interventions. This review has important implications for health care providers, intervention planners, and researchers.

## Background

Breast cancer is the most common cancer in women worldwide [1, 2]. Each year, more than 1.5 million women worldwide are diagnosed with breast cancer and 502,000 die from this disease [2]. According to the Qatar National Cancer Registry, breast cancer is the most common cancer among women in the State of Qatar [3, 4]. The breast cancer incidence rate during the year 2006 was 30 per 100,000 among women in Qatar while other cancer types fell below 9 per 100,000 women [5, 6]. According to the International Agency for Research on Cancer (IARC) [7] and GLOBOCAN 2008 [8], in the Middle East the highest age-adjusted breast cancer incidence rate has been recorded in Lebanon (55.4/100,000), followed by Bahrain (49.8/100,000), Kuwait (47.7/100,000), and Jordan (47/100,000). In Gulf Cooperation Council (GCC) countries, breast cancer incidence rates are highest in Bahrain (49.8/100,000), followed by Kuwait (47.7/100,000) and Qatar (38.1/100,000). The Qatar breast cancer incidence rate of 38.1/100,000 during the years 1998–2001 is high compared to that of countries such as Saudi Arabia (22.4/100,000) and Yemen (20.8/100,000) in the same time period [3, 7]. The WHO (2006) stated that the high mortality rates of breast cancer in Middle Eastern countries were primarily due to delayed diagnosis of the disease. According to the WHO, the breast cancer mortality rate in Qatar in 2008 was 12.9/100,000 [9]. This mortality rate is higher than in other Arab peninsular countries such as Saudi Arabia (10.4/100,000) and the United Arab Emirates (10.9/100,000), which also have the lowest incidence rates of breast cancer in the Middle East [7].

These findings indicate that more emphasis on breast cancer screening (BCS) must be provided by health care workers and strategies to reinforce cancer screening need to be assumed by the health care system to increase early detection of breast cancer in Arabic women [2, 10]. Intervention strategies to promote breast cancer awareness and screening can be investigated and adopted from studies that have been conducted with Arabic women populations to ensure that future intervention programs are effective.

Screening and early detection of breast cancer are important in reducing cancer morbidity and mortality [2, 10, 11]. National cancer organizations in the United States and Canada recommend mammograms yearly [12] or every 1-to-2 years [13, 14]. The Supreme Council of Health [4] in Qatar recommends mammogram screening for women between ages 40 and 69 every 2 years. However, Bener and colleagues [6] reported that only 23.3 and 22.5 % of the women in Qatar had undergone a clinical breast examination (CBE) or a screening mammogram, respectively. A more recent study conducted in Qatar by Donnelly and colleagues [15] reported a slight increase in CBE and mammogram uptake during the years 2010–2011, 31.3 % of study participants had undergone a clinical breast examination (CBE) and 26.9 % of participants 40 years of age or older had had a mammogram. Breast cancer screening has been offered in Well Woman Clinics located in each Primary Health Care branch in Qatar for over 10 years, but there is no national population-based BCS program. Breast cancer screening in Qatar relies on women to self-present, misses many women at risk, and fails to monitor, follow-up, and evaluate clinical and diagnostic qualities and outcomes [4]. Awatif [16] and Al-Alaboud and Kurashi [17] suggested that the lack of a national standard screening program was one of the main barriers to BCS in Saudi Arabia. There is need for a comprehensive BCS initiative in Middle Eastern countries.

In the Arabic women population, positive family history, young age, higher level of education, employment, knowledge of symptoms of breast cancer, and living in an urban area were positively associated with BCS [18]. In addition, existing studies document that a physician’s recommendation is the most powerful facilitator for mammography utilization [15, 19–21]. On the other hand, psychosocial barriers to BCS for Arabic women included fear of the screening process, fear of the mammography results, feelings of embarrassment and stigmatization, fatalism [4, 18], lack of knowledge, language barriers [22, 23], perceived transportation and economic barriers [22, 24], fear of pain or discomfort from the procedure [25], competing priorities [26], and concern that breast examination might threatened cultural and religious values [27, 28]. Some of these findings are consistent with the results of our study that explored how women living in Qatar were challenged by BCS [29].

The Supreme Council of Health in Qatar [4] recognized that lack of knowledge and awareness were major barriers to BCS. The Council recommended awareness campaigns that focused on early diagnosis to eradicate myths that can lead to fatalism and stigmatization and to educate the general public about the signs and symptoms of breast cancer [4]. Qatar has designated a Breast Cancer Month—October—during which Women’s Hospital and Think Pink Qatar organize annual breast cancer awareness events and a breast cancer awareness walk.

Over the last few decades, numerous intervention strategies have been examined to promote BCS among ethnically diverse populations. A meta-analysis [30] of interventions that promoted mammography among ethnic minority women (African Americans, Hispanics, Asians, and combined ethnic samples) was conducted in the United States between the years 2000 and 2008. In the meta-analysis literature review [30], authors indicated that 23 studies met the inclusion criteria for a meta-analysis; 61 % of which employed a randomized experimental study design. Findings of the meta-analysis indicated that access-enhancing interventions such as mobile vans and reduced-cost mammograms were shown to be most effective, followed by individually-directed interventions such as one-on-one counseling, letters to invite or remind, and telephone calls [30]. Similarly, other studies have shown that access-enhancing community outreach coupled with education programs that promote breast and cervical cancer screening have been successful in enabling ethnic minority women to overcome numerous screening barriers such as lack of transportation, financial strain, and competing priorities [31–33]. These findings indicate that improving geographical and financial access and providing breast cancer and health education are essential components in designing BCS promotion programs.

Furthermore, theory-based interventions that were culturally tailored to provide educational materials that conform to cultural values, beliefs, and practices and involve key community members were shown to be more effective than non-tailored interventions [30]. This finding is similar to that of Masi et al. [34] who conducted a systematic review of the literature to identify interventions designed to enhance breast cancer screening, diagnosis, and treatment among ethnic minority women. The authors found that culturally tailored interventions that addressed financial barriers were more effective than reminder-based interventions alone [34]. These findings are supported by Magai et al. [35] who conducted a conceptual review of common psychosocial factors influencing BCS adherence. The authors suggested that psychosocial factors such as cognitive variables (beliefs, attitudes, perceived risk and knowledge of breast cancer and mammogram screening) and socioemotional variables (social relations and support, emotional affects toward BCS such as fear/anxiety/embarrassment, emotions regulation styles such as denial) play a critical role in BCS adherence. Magai et al. [35] suggested the importance of addressing psychosocial factors along with epidemiologic and structural variables in planning an effective BCS intervention. In addition, in a systemic review of randomized controlled trials using community health workers, Gibbons and Tyus [36] suggested that Community Health Worker interventions (also known as Train-the-Trainer) were associated with a significant increase in mammography uptake rates. Culturally tailored interventions that target culture-specific psychosocial barriers, delivered by key community members, would be an important consideration when planning a BCS intervention.

Another meta-analysis conducted in the United States focused on improving mammography rates in diverse populations; participants had a high school education or less, received a low income, were members of an ethnic minority group, were more than 60 years of age, or resided in rural or inner city areas [37]. Thirty-eight studies were identified, 24 of which reported on women of color— the majority of participants being African American. Legler and colleagues [37] found that access-enhancing interventions performed better (20 % increase in mammogram use on an average) than other types of intervention; individually-directed interventions achieved a 17 % increase in mammogram use. Furthermore, the authors found that a combination of access-enhancing and individually-directed strategies realised a 27 % increase in mammogram use. However, as this meta-analysis [37] did not specify specific ethnic groups, and the majority of participants were African American, the applicability of the findings to the Arabic population is fairly limited.

Previously published literature reviews of interventions [30, 34, 37] that aimed to enhance BCS examined the largest ethnic groups in the United States (African American and Hispanic) and other minority groups with historically lower rates of BCS. To our knowledge, there is no systematic literature review of intervention studies that intended to promote BCS in Arabic women living in Arabic countries or in Western mainstream societies. This is perhaps due to the scant number of interventions conducted among Arab populations. Moreover, in the United States, Arab Americans are often grouped with Caucasians according to the United States Office of Management and Budget; therefore, accessing detailed data on the Arabic population is difficult [23, 38, 39]. The present review updates current knowledge on the effectiveness of existing interventions designed to increase BCS in Arabic populations in Arabic countries and North America and aims to improve the development of effective intervention programs that promote BCS among Arabic women living in Qatar. We address the following question: What interventions are effective in increasing breast cancer knowledge and breast cancer screening rates in Arabic populations in Arabic countries and North America?

## Methods

### Search Engines and Key Words

Following the method for systematic review outlined by Polit and Beck [40], CINAHL, Medline, Social Work Abstracts, SocINDEX, Cochrane Central Register of Controlled Trials, and Middle Eastern & Central Asian Studies databases were searched using the following key words contained in the title and abstract, relevant MeSH headings, and their combinations: (Arab* OR Muslim OR Gulf OR Islam* OR Qatar*) AND (“breast cancer” OR “breast neoplasms”) AND (screening OR “early detection” OR mammogram) AND (intervention* OR evaluation* OR education* OR awareness OR program* OR promot* OR uptake OR encourag*). Search terms were developed by a professional research librarian and two investigators and applied to the above databases by a trained masters-level graduate student. The search was repeated three times to find the highest number of articles. Two investigators then independently reviewed all located articles to confirm whether inclusion criteria were met.

### Inclusion and Exclusion Criteria

This review employed the following inclusion criteria: (1) the study provided an evaluation or description of a BCS program/educational intervention for Arabic women living in an Arabic region or in a Western multicultural society, (2) the study involved experimental, quasi-experimental, or longitudinal design, (3) the study sought to improve breast cancer knowledge and/or BCS rates in Arabic women. We defined Arabic women as women who speak Arabic and who live in Arab countries in the Middle East or North Africa, or Arab Americans and their descendants [38, 39]. Exclusion criteria were studies that evaluated breast self-examinations, nonintervention studies, biomedical/treatment research, pharmaceutical research, descriptive research, instrumental research, and studies that did not report valid outcome measures. Valid outcome measures were defined as completion of mammograms either by self-report and/or verified by a clinical record and an increase in knowledge about breast cancer and cancer screening measures by self-report.

The search was limited to the following studies: (1) written in English; (2) published; (3) peer-reviewed to assure a high level of quality of evidence and endorse validity of the overall findings and conclusions. Databases were searched with no restriction on the start date until June 2012 due to limited numbers of published studies of Arabic women.

### Search Outcome

**Fig. 1:**
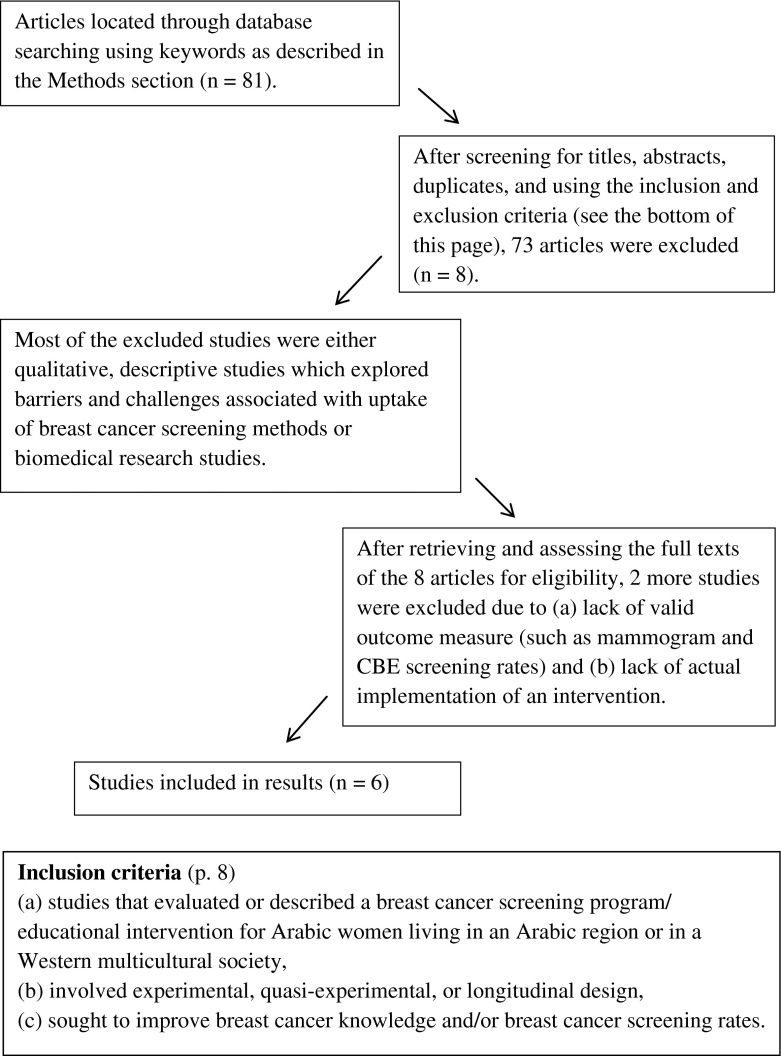
Flow diagram of literature search

A total of 81 studies were located as the result of the search—30 from CINAHL, Social Work Abstracts, SocINDEX, and Middle Eastern & Central Asian Studies and 51 from Medline (Ovid) & Cochrane Central Register of Controlled Trials. After screening the titles and abstracts of the studies brought up by the search, 73 studies were excluded from the review because they did not meet the inclusion criteria or they were duplicated across databases. The most common reason for exclusion was a lack of description or evaluation of an intervention, indicating that there are not many intervention studies in this area. Most of the excluded studies were qualitative, descriptive studies that explored barriers and challenges associated with BCS and biomedical intervention studies. The full paper was retrieved for analysis in eight studies. After reading the full text articles, two studies were excluded: one did not report a valid outcome measure and another was an instrumental study. Hence, a total of six studies were included in this review. In order to present reliable evidence of intervention effectiveness, quality assessments were conducted by the investigators following the criteria described in section 6.4 of the Data Collection Checklist from the Cochrane EPOC guidelines [41] (Fig. 1).

## Results

Six studies were located in the search (see Tables 1, 2, 3). In this review, Arabic women of different nationality (Israel, Saudi Arabia, and Arab Americans from Yemen, Egypt, Morocco, Iraq, Lebanon, and other Arab countries), socioeconomic status (SES), educational level, and age (25–75 years) are represented (Table 1). Of these six studies, three were conducted in the United States, two in Israel, and one in Saudi Arabia. Sample sizes ranged from 66 to 1,429. Most interventions appraised in this review were women-focused, personal interventions tailored to each woman, delivered face-to-face or by telephone. All studies targeted personal barriers such as lack of knowledge and awareness. In addition, two studies targeted cognitive barriers such as Arab-specific cultural beliefs and practices of women, two studies targeted social discrimination and lack of cultural competence, and one study targeted lack of an organized, population-based screening program. In all of these studies, the most common type of study intervention was an educational program that delivered culturally sensitive information about breast cancer in the Arabic language; two studies [38, 42] were concerned with multilevel interventions that targeted women and health care professionals and/or systems, and one study [43] featured a public awareness campaign. Interventions were performed by telephone [44], by increasing women’s access to mammography via home visits, community outreach, and telephone calls [23, 43, 44] and by involving key members of the community [38, 39, 43]. All studies provided free mammograms with individualized assistance (i.e., help with scheduling, accompaniment to mammogram appointments, appointment reminders, follow-up, and assistance in accessing other needed services). The strategy and effectiveness of each intervention is discussed in the section related to the specific type of study.

**Table 1:** Design, sampling, and demographic information for included studies

Study	Design and control conditions (brief description of control) as well as N in each group	Sampling method	Sampling recruitment	Demographic information on sample (targeted racial, age range, education level, SES, spoken language at home, etc.)
Ayash et al. [38]	*Quasi*-*experimental, time series design* Intervention (one community): N = 597; no control group	Convenience sampling	Not reported but assumed that women voluntarily participated in the educational intervention after seeing advertisements then screened for eligibility for cancer screening	Arab American, aged ≥27, 54 % speak Arabic at home, 31 % speaking both Arabic and English, 9 % only English; education level/SES unreported
Dallo et al. [39]	*Quasi*-*experimental, time series design* Intervention (one clinic): N = 866 (377 males and 489 females; no control group	Convenience sampling	Advertised intervention via announcements and flyers posted in places where Arab Americans regularly patronize and Arabic radio and invited to participate voluntarily	Arab American, aged ≥40 (mean age 51); 52.8 % has less than high school education; 34.8 % employed; 27.4 % less than 5 years of stay in US
Akhtar et al. [43]	*Quasi*-*experimental, non*-*equivalent control group after*-*only design* Intervention (two health care sectors): N = 1766; control being the international standard and reuptake rate	Convenience sampling	Eligible women (according to the inclusion criteria) of the two health care sectors were contacted and checked against exclusion criteria, then invited to the Primary Health Care for an assessment	Saudi Arab, aged 35–60 (mean age 47); no information available on education level, SES, or spoken language at home
Arshad et al. [23]	*Quasi*-*experimental; time series design* Intervention (one group): N = 100; no control group	Not reported	Not reported	Arab American, aged 25–57 (mean age 41); other information was not reported
Cohen and Azaiza [44]	*Experimental, controlled before and after design* Intervention: N = 42; Control (no intervention, usual care) N = 24	Convenience sampling	Participants from the previous study were approached and asked to participate. Out of random sample of 300, 74 women were recruited	Israeli Arab, aged 40–65 (mean age 49); education level ranged from none to 25 years education; SES varied; 68 % Muslim and 22 % Christian; 95 % mildly—very religious
Wilf-Miron et al. [42]	*Quasi*-*experimental; non*-*equivalent control group before*-*after design* Intervention (13 Arabic branches as one group): N = 1429; control being the overall healthcare services: N = 125063 (in 126 branches)	Purposive sampling	No recruitment of participants. Data were gathered through the organizational operational database of Maccabi Healthcare Services in Israel	Israeli Arab, aged 52–74 (mean age 60); other information were not available on the database and were not reported in the article

**Table 2:** Intervention overview of included studies

Study	Name of program	Description of program	Targeted barriers	Duration of program	Geographic location of program/location of Program	Result of intervention
Ayash et al. [38]	AMBER: Arab American Breast Cancer Education and Referral Program (funded)	Bilingual patient educators (navigators) provided workshop to women using an Arabic language curriculum, along with individual-level navigation such as risk assessment, assistance, and follow up. AMBER staff conducted Arabic cultural responsiveness trainings to health care providers and staff using a community-based participatory approach	Psychosocial factors: lack of knowledge, perceptions of risks and benefits of breast cancer screening (BCS) Lack of English skills, Lack of transportation, insurance Systemic: discrimination and lack of cultural competence in the health care	Two years (2007–2009)	Brooklyn, New York, US/ Community-wide	597 women were educated in 22 workshops; 189 women were identified as being in need of assistance; 68 were screened; 1 new case of breast cancer was detected 68 % reported increased understanding of cancer screening 29 % increase in screening among Arab American women in the community 1 year after intervention
Dallo et al. [39]	N/A	A 30-min one-on-one, bilingual, educational intervention administered to each woman participant, along with physical examination and free cancer screening	Psychosocial factor: lack of knowledge and perception of benefits of BCS Lack of English skills	Two years (2005–2007)	Michigan, US/ Clinic-based	For each 12 questionnaire items that test pre- and post-intervention knowledge (see Table 3 in Dallo et al. [39]), cancer knowledge significantly increased after intervention compared to prior to the intervention, especially among the disadvantaged participants
Akhtar et al. [43]	Al-Qassim Screening Mammography Program, Population-based (funded)	Breast cancer screening program and campaigns were announced via media channels, newspapers, exhibitions, lectures, information stalls, and posters. A public awareness team held interactive educational sessions with both men and women	Psychosocial factors: lack of knowledge and awareness Lack of organized, population-based screening program	1.5 years (Jan 2007–June 2008)	Saudi Arabia/Community-wide	18 % of the total population in the two health sector areas participated in mammogram screening (lower than the international standard), with high recall rate (31.6 %)
Arshad et al. [23]	N/A	Bilingual Arab community health workers delivered the educational intervention in the homes of Arab-American women with their adult female family members	Psychosocial factor: lack of knowledge Lack of English skills	One time intervention	Michigan, US/ Community-based	The educational intervention improved women’s knowledge of BSE, CBE, and mammogram regardless of their language preference. Higher education level and younger age were significant predictors of improvement
Cohen and Azaiza [44]	Tailored culture-based, telephone intervention	A trained social worker addressed Arab culture-specific barriers by answering to the barriers and misconceptions and using religious and cultural promoters	Psychosocial factors: perceived barriers (cultural beliefs, social norms), lack of knowledge Lack of cultural competence in interventions	6 months	Israel/Intervention group-based	48 % intervention group attended CBE versus 12.5 % control group 38.5 % intervention group attended or scheduled mammography versus 21.4 % control group Intervention group perceived fewer barriers after the intervention when compared with the control group
Wilf-Miron et al. [42]	N/A (funded)	Combined macro-organizational, top-down (development of computerised system and infrastructure to reach potential, eligible women participants) and bottom-up interventions (tailored local educational programs)	Psychosocial Factors: lack of knowledge and perceived benefits of BCS, lack of self-care values, social norms, social influences, religious values ↓accessibility System—lack of effective infrastructure	Two years (2004–2005)	Israel/Community-wide	Average breast cancer screening rates in Arab branches increased from 26.7 to 46.2 % (73 % improvement). Reached 80 % of the women eligible for breast cancer screening

**Table 3:** Methodological quality of included studies

Study	Allocation methods	Attrition	Other potential bias
Ayash et al. [38]	Lack of randomization	High attrition rate	Baseline data are not reported Hawthorne effect Effects of external factors
Dallo et al. [39]	Lack of randomization	Low attrition rate	Desirable response bias Response shift bias
Akhtar et al. [43]	Lack of randomization	High attrition rate	Baseline data unavailable Number of participants in the interaction sessions and how successful the collaboration among the community members were not reported
Arshad et al. [23]	Lack of randomization	No attrition	Selection bias Training process of the community health workers was not reported. Accuracy of information and confidence in the manner that educational intervention was delivered were not documented
Cohen and Azaiza [44]	Random allocation met	Low attrition rate	Lack of allocation concealment Desirable response bias Small sample size
Wilf-Miron et al. [42]	Lack of randomization	N/A	The extent of spillover effect is unclear

The methodology of each study is presented in Table 1, descriptions of the interventions are offered in Table 2, and the methodological quality of each study is outlined in Table 3. Five of the six studies employed quasi-experimental design, where treatment and control groups were chosen by convenience rather than by random selection. Of these five studies, four studies did not have control groups which could have strengthened the validity of the findings. Only one study by Cohen and Azaiza [44] employed a randomized, controlled trial design, but it had a small sample size and randomization occurred at the individual level rather than at the cluster level. The quality appraisal of the studies included in this review suggests that future intervention or evaluation studies should employ vigorous study designs. Nevertheless, these studies provide evidence of the effectiveness of different intervention strategies in increasing BCS rates in Arabic women populations.

### Description and Effectiveness of Various Interventions Strategies

#### Interventions Tackling Psychosocial Factors

Lack of knowledge about breast cancer, one of common psychosocial barriers [35, 45], was the main barrier to be identified and addressed in all the studies reviewed. Therefore, education about breast cancer and cancer screening methods was a part of all interventions. Although the various intervention types approached the barrier differently, all of the studies stressed the importance of delivering educational material via the Arabic language using a bilingual health educator, addressing women clients’ perceptions of Arab-specific cultural norms, and delivering culturally sensitive information.

##### Intervention Strategies Using the Arabic Language and Bilingual Educators

Three of the studies included in this analysis were conducted in the United State [23, 38, 39]. All identified limited language skills contributing to women’s lack of appropriate knowledge of BCS. These studies delivered breast cancer education in the Arabic language using bilingual health educators (lay health navigators) or Arab community health workers (CHWs).

Drawing on research findings from other Arab American studies and focus groups with Arabic women, Ayash et al. [38] created the Arab American breast cancer education and referral (AMBER) Program to tackle the lack of knowledge and perception of risks of breast cancer and benefits of BCS. The curriculum developed in Arabic included information about breast cancer, cancer risk reduction, early cancer detection methods, and community resources. The mode of intervention was via individual-level counseling and navigation. The result of this intervention was that 68 % of the participants self-reported an increased understanding of cancer screening and 36 % of the participants undertook mammogram screening. The authors also reported that there was a 29 % increase in the screening rate among Arab American women in the target community 1 year after the intervention. However, generalization of their findings is limited by the geographic location (United States), the high attrition rate of study participants, and a lack of baseline data needed to compare post-intervention behavior with pre-intervention behavior. Furthermore, as this intervention targeted health care professionals, the effects of external variables that could have affected women’s participation in BCS need to be considered. Ayash et al. [38] tackled emotional barriers such as fear, feelings of being discriminated, and women’s perception of health care providers by providing cultural responsiveness training to the staff. The result that shown 36 % of the participants undertook mammogram screening suggested effectiveness of the cultural responsiveness training in addressing emotional barriers.

The interventions reported in Arshad et al. [23] and Dallo et al. [39] were solely educational, tackling lack of knowledge as the major barrier to BCS. Both were conducted in Michigan where many Arab Americans settled. The educational contents of the intervention included types and most common forms of breast cancer, risk factors, and the importance of screening, early detection, prevention, risk-reduction strategies, and treatment of cancer [23, 39]; the curriculum was developed based on sources such as the Centers for Disease Control and Prevention, the National Cancer Institute, and Cancer Control P.L.A.N.E.T. [39]. Both interventions were delivered bilingually. The interventions reported in Dallo et al. [39] were delivered face-to-face with individual women for 30 min in the clinic and involved discussions of cancer prevention and risk reduction strategies such as changes in diet, physical activity, and smoking cessation, as well as physical examination and annual mammogram screening. Arshad et al. [23] reported that interventions were held in the women’s homes with a small group of adult female family members present. The authors of both studies [23, 39] reported significant improvements in participants’ knowledge of breast cancer and cancer screening methods post-intervention compared to pre-intervention. Dallo et al. [39] also reported a significant increase in perceived importance of BCS after intervention, suggesting a well-planned and implemented education intervention might change perceptions about BCS (perceived risks and benefits), thereby influencing adherence to BCS guidelines.

The authors of both studies concluded that consideration of the women’s educational status was crucial to planning such an intervention program. However, Dallo and colleagues’ interventions [39] were most effective for disadvantaged populations with low SES and education levels while Arshad and colleagues’ interventions [23] were more effective for people with higher education than those with lower education. This inconsistency in findings could be due to methodological factors such as different sample size, sample characteristics, and sampling methods (see Table 1); different intervention venue (i.e., home vs. clinic); and other potential biases (see Table 3). Despite the success of both studies [23, 39] in improving the target population’s breast cancer knowledge, the authors did not assess mammogram screening rates pre- and post-intervention. Thus there is no indication of whether there was an increase or a decrease or no change in mammogram screening rate which is an ultimate goal of BCS interventions.

##### Intervention Strategies that Deliver Culturally Sensitive Information

While studies conducted in the United States identified lack of English skills as the main barrier to acquiring information about breast cancer, the two interventions in our review that were performed in Israel [42, 44] targeted women’s psychosocial barriers and lack of cultural competence in screening programs as the main barriers to delivering breast cancer education effectively.

Breast cancer education interventions reported in Cohen and Azaiza [44] and Wilf-Miron et al. [42] involved addressing sociocultural norms, moral values, and misconceptions, and delivering culturally sensitive information about breast cancer. For example, a tailored, culture-based, telephone intervention basing its foundation on the health belief model and the transtheoretical model [44] was performed by a trained social worker with a nursing education background. The interviewer asked about the woman participant’s previous experience with breast examinations and her perceived beliefs and barriers to BCS. Using a cultural competency approach, issues were addressed regarding misconceptions about breast cancer and early detection, and cultural barriers experienced by many ethnic women (i.e., exposure of the body, social barriers, religious beliefs about cancer and health, environmental barriers, and uneasiness with one’s own body). The interviewer also emphasized cultural and religious reasons for health preservation and explained the mammogram procedure in terms chosen to allay anxiety (the pain is mild, lasts for only a few seconds). The scripted answers to cultural barriers and a list of religious and cultural promoters were prepared based on five focus groups previously conducted with Arabic women. This culture-based intervention significantly raised the number of women in the intervention group who underwent clinical breast examinations (CBE) and mammograms compared to the control group (CBE: 48 % intervention group vs. 12.5 % control group; mammogram: 38.5 % intervention group vs. 21.4 % control group). The authors reported that the intervention group perceived fewer barriers to CBEs and mammograms, had a higher perception of personal susceptibility to breast cancer, and recognized more benefits of CBE compared with the control group [44]. Nonetheless, small sample size and other potential biases such as lack of allocation concealment and desirable response bias limit the generalizability of the findings (see Tables 1, 3.). Allocation concealment can be performed in any type of random assignment [40]; however, Cohen and Azaiza [44] did not report in their article neither randomization procedure nor use of allocation concealment. Desirable response bias can occur in self-reports, where respondents may provide biased responses reflecting perceived expectations [40].

Wilf-Miron et al. [42] identified specific psychosocial barriers such as lack of knowledge and perceived benefits of BCS, perceived social norms (family disapproval of women leaving the city unaccompanied by a male relative), moral/religious values (embarrassment with a male physician conducting a breast examination), and social influences (family/friend disapproval of the screening), and low levels of self-care values. These barriers were addressed using community leaders to explain the benefits and importance of early detection, how examinations are scheduled, and how examinations are performed. However, how the community leaders addressed other identified psychosocial barriers such as religious values and social influences and persuaded women to participate in BCS is not reported in detail. Wilf-Miron et al. [42] reported an average improvement of 73 % in the BCS rate in Arab branches of the breast screening clinics. However, this intervention was part of a larger, multilevel intervention that included system targeted and access-enhancing interventions (see “Multi-level Interventions” in the following section); therefore, this success cannot be attributed solely to culturally sensitive education.

#### Multi-level Interventions

Two intervention studies, one with Arab Americans in the United States [38] and one with Arabs in Israel [42] targeted women, health care providers, and the health care system in the interventions. However, the target barriers were slightly different with each study. Lack of effective health care infrastructure was a systemic barrier according to Wilf-Miron et al. [42], whereas social discrimination and lack of cultural competence in the breast screening centers were identified as systemic barriers in Ayash et al. [38].

##### Targeting Health Care Professionals

The AMBER project by Ayash et al. [38] addressed breast health disparities experienced by Arab Americans through a multilevel approach that facilitated women’s breast cancer knowledge and awareness (see “Intervention strategies using the Arabic language and bilingual educators”) and the readiness of the health care system to serve Arab American breast health clients. The intervention was given in the form of cultural responsiveness training at health care facilities located in Arab American communities; trainees included health care providers and all clinic staff. The curriculum was developed through focus groups with 27 staff members prior to the intervention. The contents of the curriculum included working with interpreters, conducting cross-cultural medical interviews, addressing healthcare seeking behaviors and cancer services access patterns in the Arab American community, and strategies to overcome linguistic, economic, legal, and cultural barriers to BCS. Group exercises involved debunking common stereotypes, including origins, practices, and traditions in the Arabic population, and presentations concerning culturally-specific health-related beliefs and practices, language, styles of dress, and historical perspectives of various Arabic populations. Ayash et al. [38] reported that the intervention was effective with a 29 % increase in screening among Arab American in the target community 1 year after the intervention. However, the authors suggest that the findings should be understood with consideration of methodological limitations and effects of external factors that might have affected the screening rate in the community.

##### Targeting the Health Care System

A multilevel intervention that combined macro-organizational and tailored local programs approaches to facilitate women’s breast cancer knowledge and the health care system’s solid infrastructure on which local initiatives could grow was exemplified in Wilf-Miron et al. [42] (also see “Intervention strategies that deliver culturally sensitive information”). The computerized system enabled monthly scanning of the population to construct a list of members requiring mammogram screening and delivery of screening invitation cards. Non respondents to invitations were identified at the branch level and contacted by phone. Computerized decision-support tools were developed to notify the primary physician as to whether the patient had undergone screening. Flexibility was introduced into the clinical protocol to meet patient needs, i.e., the male physician’s CBE could be waived if it caused distress. Physicians received information comparing their patients’ screening rate with regional and organizational rates for peer comparison and motivation. However, the authors suggested that this particular strategy did not work because dialogues between managers and physicians following these reports did not take place. An access-enhancement strategy was also an integral part of this intervention with local branch staff organized group transportation to the screening facility, and the use of a mobile screening facility that went door-to-door. This combined top-down and bottom-up approach was found to be the most effective among the appraised studies with 73 % improvement in the BCS rates in Arab branches (see “Intervention strategies that deliver culturally sensitive information”).

#### Public Awareness Campaign

The Al-Qassim Screening Mammography Program conducted in Saudi Arabia by Akhtar et al. [43] involved a public awareness campaign as a main strategy to increase the BCS rate. The target barriers were lack of knowledge and awareness (psychosocial barrier) and lack of an organized, population-based screening program (systemic barrier). Breast cancer facts, health information, and information about an available BCS program were disseminated through media channels, newspapers, exhibitions, lectures, posters, information stalls in shopping malls, and board and banner displays in visible areas of the region over 2 years. A public awareness team consisting of community medical experts, female Saudi Arabian nurses and social workers, and a committed group of female volunteers held public, interactive, educational sessions with women to discuss the importance of early detection of breast cancer and to describe breast screening methods. Importantly, the male members of the public awareness team held similar sessions for men to raise men’s breast cancer awareness and understanding of their female partners’ need to have CBEs or mammograms. However, information about how the interactive, educational session was facilitated and what strategies were incorporated to improve perceived importance and benefits of BCS were not reported.

After these community-wide public awareness campaigns, eligible women listed in the Public Health Care (PHC) database were contacted and invited to the PHC centers. Despite these intensive public awareness efforts and personal invitations to screening, results of the intervention were discouraging. Akhtar et al. [43] reported that only 18 % of the total population in the target areas participated in mammogram screening, a percentage lower than international standards (>75 % both in the EU and the UK), with a 31.6 % recall rate that was much higher than international standards (<5 % for the EU and <7 % for the UK). Thus, it was suggested that the intervention strategies were ineffective and costly and did not increase the mammogram participation rate. However, it is difficult to determine the effectiveness of this program as there was no baseline data for comparison. Although the screening rate was much lower than international rates, the post-intervention screening rate of the target region might have increased compared to the pre-intervention rate. Use of a quasi-experimental, time series design with a control group would have measured the effectiveness of the intervention. Moreover, information about the number of participants in the interaction sessions and the degree of community collaboration could have assisted in interpreting the study findings.

### Recommendations from Studies’ Authors

The recommendations offered by the authors in research studies appraised for this review can be summarized as the following: (a) educate the women and (b) tailor breast cancer screening interventions to the population’s unique needs.

#### Educate the Women

The significance of breast cancer education was underscored in all of the appraised studies. For example, Arshad et al. [23] showed that education was most effective for women with higher education and younger age. The authors recommended that young women be educated about breast health before the age of recommended BCS. They suggested that early education could have a positive impact when women reach the age of screening and stressed the importance of presenting accurate information to the public so that the education does not misrepresent BCS. On the other hand, Dallo et al. [39] suggested that the intervention was most effective among disadvantaged participants, inferring the importance of reaching out to disadvantaged populations to improve the BCS rates. In Cohen and Azaiza’s study [44], the use of tailored, educational intervention strategies that targeted cognitive psychosocial barriers proved to be effective on health-related behavior change; the authors recommended that intervention should be interactional, motivational, and perception-changing, rather than being informative and instructive.

#### Tailor Breast Cancer Screening Interventions to the Population’s Unique Needs

Most researchers agree that tailoring an intervention to a population’s educational, language, cultural, SES, employment, and health behavioral characteristics will increase its effectiveness. Cohen and Azaiza [44] concluded that the cultural, social, and historical context of Arab societies greatly influences the beliefs, attitudes, and behaviors of Arab women toward BCS. Thus it is important to develop culture/place-specific interventions that address perceptions of sociocultural norms, social influences, and beliefs of the ethnic group involved in the intervention. Pilot intervention studies in Cohen and Azaiza [44] and Wilf-Miron et al. [42] used focus groups to ascertain the local population’s barriers to accessing cancer screening services. Both studies effectively decreased perceived psychosocial barriers to CBEs and mammograms and increased screening rates [42, 44]; these results infer the importance of tailored, culture-specific, interventions.

## Discussion

This systematic review of six research studies that focused on Arabic women’s breast health behaviors presents an overview of strategies for BCS interventions and evaluates their effectiveness. The intervention studies varied in geographic area, study population, and target barriers. Unfortunately, there were too few studies to test whether or how the type of intervention employed impacted the findings. Intervention effectiveness was found to be dependent on the study setting, the historical, cultural, and demographical contexts of participants and their beliefs and attitudes about breast cancer and cancer screening, and the intervention methods employed.

We infer several insights from this review. First, a language-appropriate and culturally sensitive (culture/place-specific) educational program is the most important component of a successful intervention to address psychosocial barriers, the disparity in knowledge and attitudes experienced by Arabic women, regardless of the study setting. Second, multi-level interventions that target general populations (especially women populations), health care professionals, and/or larger systems are more likely to be successful than single educational interventions or public awareness campaigns. Third, more vigorous, personal (face-to-face, by telephone), and cognitive interventions are likely to be more effective than less personal (media campaigns, invitational letters) and informative interventions.

Psychosocial factors are reported to have a strong influence on health protective and health maintenance behaviors and are very widely studied in cancer screening literature [35, 45, 46, 47]. The articles appraised in this review identified and addressed various psychosocial barriers such as lack of knowledge, lack of perceived risks of breast cancer and benefits of BCS, lack of self-care values, perceived vulnerability, emotions such as fear, anxiety, and embarrassment, perceived discrimination from health care providers, sociocultural health beliefs, perceived social norms, and social influences (See Table 2). Researchers and intervention planners should take these factors into account when designing future BCS interventions and be mindful to report in details strategies used to ameliorate the barriers. None of the appraised studies embraced the term “psychosocial factors.” Instead, many factors were referred to as cultural barriers. Aside from addressing cultural beliefs and practices, intervention targeting modifiable cognitive beliefs, perceptions, and emotional experiences pertaining to BCS would provide an operational means to tackle complex psychosocial barriers. It is encouraged that future intervention research using a bottom-up approach employ theoretical frameworks that address psychosocial barriers such as an expanded version of Andersen model [48, 49, 50]. Addressing various psychosocial factors along with epidemiologic and structural factors might contribute to differences in BCS rates.

Less evident insights that emerged during analysis of the studies suggested factors that can improve BCS rates in the Arabic population. (1) Offer free mammograms to ease the financial burden of screening services. All studies included in the review offered free mammograms and CBEs. (2) Increase the geographical area of the interventions to reach more women. Although home outreach, group transportation services, mobile screening facilities, and transit passes were not directly examined as independent variables, all studies assisted women participants to gain access to available screening services. (3) Employ a support person/liaison. Almost all studies attended to the socio-emotional barrier (embarrassment engendered by a male physician conducting a CBE) by including female health personnel and assigning a female support person as a patient navigator. Patient navigators assisted with making appointments, calling women to encourage them to make appointments, ensuring that women had received mammogram results, accompanying the women to their appointments, and assisting them with interpretation, billing issues, and necessary follow-up care [38, 39]. Furthermore, associated with the recognition of breast cancer as an issue, and barriers to early detection by engaging in breast examinations, are cultural attitudes toward gender and sexuality among both women and men. Breast examinations require some degree of openness about examining of the women’s body by either male or female examiners. In some cultures, discussion of breast and its examinations is considered taboo because it is associated with sexuality and breast cancer creates social stigma for women [15, 29]. Bener and colleagues [6, 18] found that in some conservative Arab areas, access to mammography clinics may be a barrier if women are not allowed to drive or travel alone without a male. Thus women are particularly vulnerable to the risk of discovering breast cancer at the late stages if they are not comfortable raising issues of breast lumps and breast examinations, especially, if their male relatives are not supportive of or object to such examinations. The above information emphasizes the importance of including men in health promotion messages about BCS, so that they can encourage and support their wives’ and female relatives’ decision to go for mammography. It points to the realization that to effectively reduce breast cancer’s morbidity and mortality rates by early detection, we need to promote BCS activities in ways that are culturally appropriate and acceptable to not only Arab women, but also Arab men. (4) Involve community leaders and key organizations in the intervention. Many interventions were community-based; therefore, community collaboration was central to the intervention. Ayash et al. [38] and Akhtar et al. [43] documented that established relationships and trust in community leaders, government officials, teachers, clergy, and other persons of social importance from community-based organizations, mosques, and churches facilitated smooth intervention entry to the community, increasing the likelihood of success. Although ethnicity varied, the findings in this review of Arab women are consistent with the findings in literature reviews of research studies of other ethnic women [30, 34, 37].

Except for one study, all interventions were found to be effective either in improving cancer knowledge or increasing CBE/mammogram screening. However, as discussed for each study in the “Results” section, effectiveness of the intervention needs to be appraised critically, considering methodological strengths and weaknesses, and potential biases that might have affected the findings. Even for meticulously designed studies, some biases are inevitable. The overall quality of included studies is moderate. Limitations of studies appraised in the review included lack of control and randomization, lack of baseline data, small sample size, high attrition rate, and other potential biases such as the Hawthorn effect and the desirable response bias. These limitations in methodology of appraised studies warrant further intervention studies that employ meticulous experimental and quasi-experimental designs and probability sampling methods that avoid biases more effectively. Furthermore, to improve the evidence base, large-scale studies with longer follow-up periods are needed to strengthen the credibility and validity of study results that will be used to plan future interventions. Use of a prospective, longitudinal design will also enable researchers to assess the sustainability of interventions. Finally, future studies must assess the increase in the mammogram screening rate as an outcome measurement of the intervention, as it is the ultimate aim of these BCS promotion interventions.

This review has implications for health care providers and intervention planners. It is important to recognize that Arabic women have disparate levels of knowledge of and attitudes toward breast cancer regardless of the setting, education level, and SES status. Physicians and nurses can improve BCS by educating women about breast cancer and encouraging screening while acknowledging culturally-specific beliefs and practices and avoiding stereotyping. Social workers and community liaisons can bring women and health care systems together.

## Limitations

Although we did not limit our search criteria to publication year and full-text articles, we were able to locate only recent articles with full-text. This probably reflects a shortage of breast cancer intervention studies in Arab populations. We did not search non-English research articles, gray literature, or unpublished studies, and might have overlooked studies with potential value. The credibility and strength of the results of a meta-analysis depends on the primary studies that comprise it. As discussed earlier, more than half of the primary studies did not involve randomization and/or control. Given methodological limitations of non-probability sampling and quasi-experimental design, researchers are strongly encouraged to explore alternative strategies to alleviate weakness and bias and provide methodological rigor in future studies.

## Conclusion

This review demonstrated the effectiveness and applicability of existing interventions to promote breast cancer screening in Arabic populations. We identified six themes: (1) provide language-appropriate and culturally sensitive educational programs to women; (2) employ multilevel interventions to maximize the synergic effect between each level of intervention; (3) deliver interventions that explore cognitive barriers personally (face-to-face or on the telephone); (4) offer free mammograms; (5) incorporate access-enhancing strategies; and (6) employ a support person (also called liaison or patient navigator) who can act as a mediator between women and the health care system and provide support and encouragement. Many more intervention and evaluation studies are needed in this area to develop culturally sensitive interventions and assess the cost-effectiveness and long-term sustainability of the programs.
